# Dietary raisin intake has limited effect on gut microbiota composition in adult volunteers

**DOI:** 10.1186/s12937-019-0439-1

**Published:** 2019-03-07

**Authors:** Akemi T. Wijayabahu, Sheldon G. Waugh, Maria Ukhanova, Volker Mai

**Affiliations:** 1 Department of Epidemiology, College of Public Health and Health Professions and College of Medicine, University of Florida, Gainesville, USA; 2 Emerging Pathogen Institute, University of Florida, Gainesville, USA; 3 Present address: Department of Epidemiology, College of Public Health and Health Professions and College of Medicine, Emerging Pathogen Institute, University of Florida, 2055 Mowry Road, Room 373, Gainesville, Florida, 32610-0009 USA

**Keywords:** Human, Gut microbiota, Sun dried raisins, Healthy adults

## Abstract

**Background:**

Dried fruits, such as raisins, contain phytochemicals and dietary fibers that contribute to maintaining health, potentially at least partially through modification in gut microbiota composition and activities. However, the effects of raisin consumption on gut microbiota have not previously been thoroughly investigated in humans. Therefore, the objective of this study was to determine how adding three servings of sun dried raisin/day to the diet of healthy volunteers affects gut microbiota composition.

**Methods:**

A 14-day exploratory feeding study was conducted with thirteen healthy individuals between the ages of 18 and 59 years. Participants consumed three servings (28.3 g each) of sun dried raisins daily. Fecal samples were collected prior to raisin consumption (baseline) and after the addition of raisins to the diet (on days 7 and 14). To determine the effects of raisin intake, fecal microbiota composition before and after raisin consumption was characterized for each participant by 16S rRNA gene sequencing.

**Results:**

Overall microbiota diversity was not significantly affected by adding raisins to the diet. However, upon addition of raisins to the diet specific OTUs matching *Faecalibacterium prausnitzii*, *Bacteroidetes sp.* and *Ruminococcus sp.* increased in prevalence while OTUs closest to *Klebsiella sp.*, *Prevotella sp*. and *Bifidobacterium spp*. decreased.

**Conclusion:**

Our findings suggest that adding raisins to the diet can affect the prevalence of specific bacterial taxa. Potential health benefits of the observed microbiota changes should be determined in future studies in populations for which specific health outcomes can be targeted.

**Trial registration:**

http://www.clinicaltrials.gov; Identifier: NCT02713165.

**Electronic supplementary material:**

The online version of this article (10.1186/s12937-019-0439-1) contains supplementary material, which is available to authorized users.

## Background

The human gut harbors a diverse collection of microbes, frequently referred to as gut microbiota, that interact with the host in a mostly symbiotic manner. The interplay between gut microbiota, diet, and health has received increased research interest over the past two decades [1, 2] and revealed multiple interactions that might contribute to maintaining good health [3–5]. Gut microbiota mediated dietary fiber fermentation generates short chain fatty acids (SCFAs) that have been correlated with health benefits [1, 3, 6–9]. The intake of raisins has been associated with i) reduced risk of coronary heart disease, ii) reduced risk of metabolic syndrome, iii) improved bowel function and iv) reduced bile acid levels [10–12]. In comparison to fresh grapes raisins are enriched in phytochemicals such as phenolic acids and tartaric acid as well as fermentable fibers such as inulin-type fructans that affect gut microbiota composition [13–15]. Based on an estimated annual consumption of 205, 000 metric tons [16] the average per person raisin intake in the US currently is only 635 g/year or less than 2 g/day.

Adding grapes or grape derived products (raisin extracts or grape pomace) to the diet has been shown to exert potentially beneficial changes in gut microbiota of mice, chicken, lambs and weaning pigs [17–20]. Two studies have shown anti-inflammatory effect of raisins in a human colon cancer cell line [21] and a human gastrointestinal epithelial cell line [22]. However, only a few studies have investigated effects of raisins on gut microbiota. Incubation of sun dried raisins with human fecal samples in a gastrointestinal model changed microbiota composition within 24 h [15]. Thus, there is a need to determine the effects of raisins on gut microbiota and establish potential health benefits in humans.

In this study we investigated how the addition of raisins to the normal diet of healthy volunteers changed their gut microbiota composition. Based on the previous literature we hypothesized that raisin intake could significantly change the gut microbiota composition of adults as compared to the baseline composition.

## Methods

### Study design

#### Participant recruitment criteria

We recruited potential study participants at the University of Florida between January 2016 to March 2016 and enrolled individuals that were between 18 and 75 years old, in general good health and had bowel movements at least three times a week. We excluded potential participants who i) had underlying digestive disorders such as gastric ulcers, Irritable Bowel Syndrome, lactose intolerance, chronic constipation or diarrhea, ii) had changes in body weight of more than 10% in the last three months and iii) had taken medication that affects bowel function or microbiota, such as laxatives and antibiotics, within the past month.

Eighteen participants were initially enrolled. Participants provided information on their demographics, general health and physical activity pattern. At each sample collection participants provided gastrointestinal health information using a questionnaire. Thirteen participants completed the study protocol and were included in the final analysis (Additional file 1: Figure S1).

#### Dietary intervention

The participants were asked not to consume any supplements or foods that could affect bowel function or microbiota (probiotics/prebiotics, digestion cleansing products, stimulant laxatives, bile acid stimulants) and to limit the alcohol intake to one drink per day.

Each participant was asked to consume three servings of prepackaged sun dried raisins (Sun-Maid Growers California) per day for a period of 14 continuous days. One serving contained 28.3 g raisins and provided 90 cal and 2 g of dietary fiber. Compliance to the study protocol was assessed on days 7 and day 14 by questioning participants about their adherence.

#### Fecal sample collection

The participants provided the first fecal sample on the day before start of raisin consumption (baseline), and after one week (day 7) and two weeks (day 14) of raisin consumption. The fecal samples were transported to the lab within 6 h of defecation and immediately processed and stored at − 20 °C until DNA extraction.

#### DNA extraction and PCR amplification

DNA from 39 fecal samples (three samples from each individual) was isolated using a modified Qiagen fecal DNA extraction protocol with an initial bead beating step as previously described [23]. DNA samples were amplified using bar-coded Illumina primers targeting the V1 and V2 region of the bacterial 16S rRNA gene (universal primer set 27F and 533R was used).

#### DNA sequencing and clustering into operational taxonomic units (OTUs)

Samples were pooled by combining equimolar amounts of 39 distinct PCR products for multiplexed sequencing. Amplicons were sequenced using the Illumina MiSeq platform. From the resulting raw data, sequences of low quality (USEARCH quality filter and chimera detection) or with a paired read length less than 290 nucleotides were removed from the analysis. Using a modified UPARSE algorithm, the sequences were clustered into Operational Taxonomic Units (OTUs) at similarity levels of 95 and 98%. A representative sequence from each OTU was annotated through the “Greengenes” 16S rRNA gene reference database using a Bayesian RDP classifier [24].

#### Data analysis

We compared the gut microbiota composition (core diversity measures, relative abundance of bacterial phyla and prevalence of individual OTUs) of fecal samples provided by each participant at week 1 and week 2 (after raisin intake) with the gut microbiota composition at baseline to identify changes after raisin intake.

Core diversity measures, the Chao1 rarefaction curve for alpha diversity and principal component analysis plots of weighted UniFrac distances for beta diversity were generated using the QIIME software package and the R package *phyloseq* [25, 26]. We also calculated Shannon-Weaver and Simpson index (alpha diversity indexes) and mean UniFrac distances.

We calculated the percent relative abundance of bacterial phyla, by combining OTUs with the same phylum level taxonomic classification into the corresponding phylum group. OTUs annotated as “un-classified” or classified only up to the kingdom level were manually aligned using BLAST [27]. Sequences matching to phages or vectors, and/or sequences with a similarity score and query coverage less than 95% were excluded from the analysis.

The significance of differences in the proportion of participants showing the presence/absence of specific OTUs was calculated using the z-test (*p*-value < 0.05). Heat maps were generated to include all OTUs that reached significance [28]. The significance in mean counts of OTUs was calculated by the t-test (p-value < 0.05). Due to the exploratory character of our study and the small sample size we did not adjust significance levels for the multiple analyses that were performed.

## Results

### Participant characteristics

The thirteen participants were between 18 and 59 years old; 62% of the participants were females, 53% overweight or obese (BMI ≥ 25.0 kgm^− 2^), 54% indicated moderate to vigorous physical activity levels. The participants did not report any discomfort (gastrointestinal health) during the raisin feeding period. However, multiple participants reported difficulties with consuming the relatively large amount of three 28.3 g servings of raisins daily for a continuous period of 14-days.

### Sequencing output

16S rRNA gene sequencing using Illumina MiSeq platform generated a total of 5,533,527 sequence reads. After removal of low quality and short length sequences, a total of 4,477,275 sequences with an average paired sequence length of 322.25 nucleotides were retained. Binning at 95 and 98% similarity levels resulted in 1238 and 2168 unique OTUs respectively. After removal of singletons we retained an average of 106,475 sequences/sample at the 95% similarity level and 103,653 sequences/sample at the 98% similarity level.

### Core diversity analysis

The Chao1 rarefaction curve suggests that we reached sufficient sequencing depth and that OTU richness did not differ significantly between any of the three time-periods (Additional file 2: Figure S2). Raisin consumption did not affect other alpha diversity measures (data not shown). UniFrac diversity analysis, a measure of beta diversity (between sample diversity) indicates that inter-individual variation was large and the impact of raisins on overall microbiota structure was limited (Additional file 3: Figure S3). Mean UniFrac distances between participants did not differ significantly at each time-period supporting the argument that raisin intake had no effect on overall microbiota composition (data not shown).

### Relative abundance of bacterial phyla

**Fig. 1:**
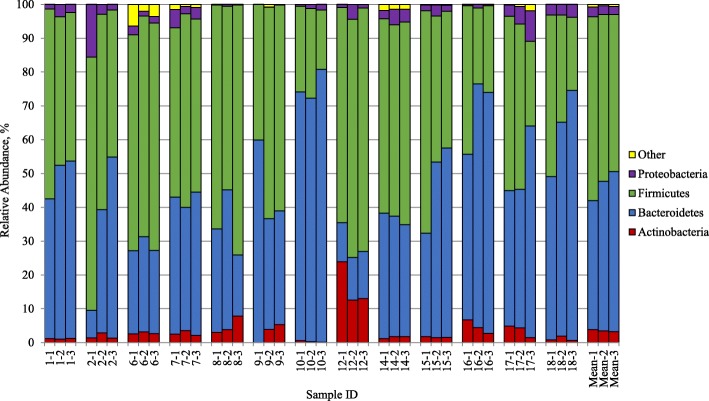
Relative abundance of bacterial phyla by sample (%, *N* = 13). OTUs that had sequence abundance mean of less than 1.0% were grouped into “Other” which included Cyanobacteria, Fusobacteria, Lentisphaerae, Synergistetes, Tenericutes, TM7 and Verrucomicrobia. Each column shows the phylum distribution (%) of gut microbiota per fecal sample. Sample ID denotes the participant ID and time-period (baseline − 1; one week after raisin intake − 2; two weeks after raisin intake − 3)

Next, we determined the effects of raisins at the phylum level; abundance was not significantly affected by raisin consumption (Fig. 1). While there were indications for a trend towards an increased relative abundance of Bacteroidetes and decreased relative abundance of Firmicutes, these observations at the phylum level did not reach statistical significance.

### OTUs significantly affected by raisin consumption

**Fig. 2:**
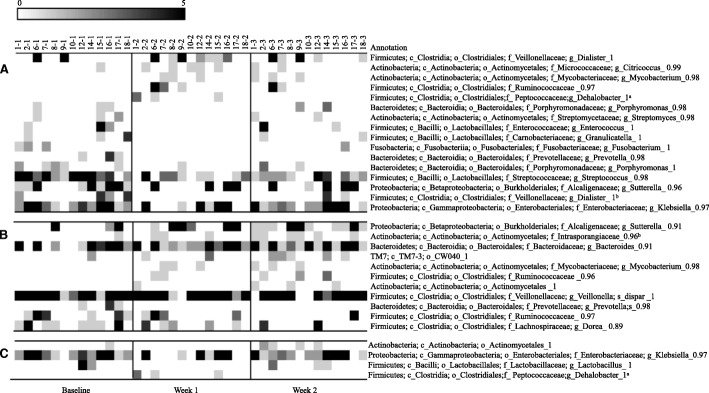
Heat-map of bacterial Operational Taxonomic Units affected by sun dried raisin intake at 95% similarity level (N = 13). Columns show samples by subject and rows show OTUs that significantly differed (*p* < 0.05) in prevalence by time period. (**a**) comparison between week 1 and Baseline, (**b**) comparison between week 2 and baseline and (**c**) comparison between week 2 and week 1 (to assess temporal effect)

**Fig. 3:**
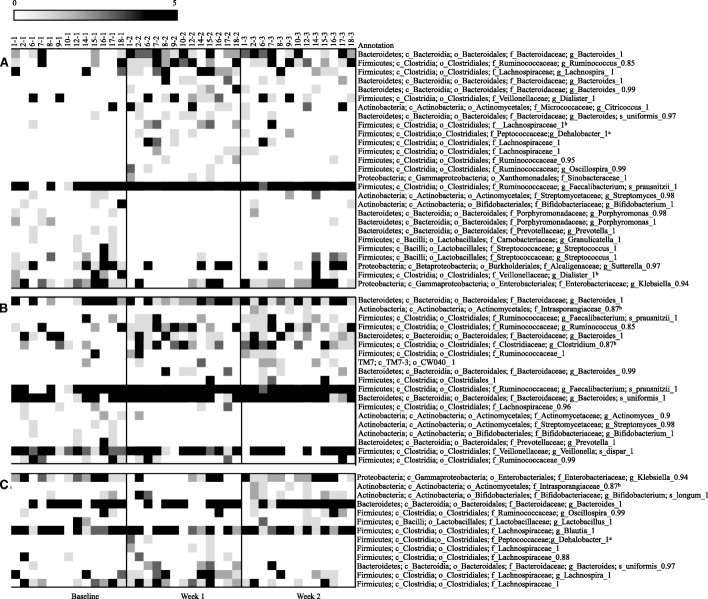
Heat-map of bacterial Operational Taxonomic Units affected by sun dried raisin intake at 98% similarity level (N = 13). Columns show samples by subject and rows show OTUs that significantly differed (p < 0.05) in prevalence by time period. (**a**) comparison between week 1 and Baseline, (**b**) comparison between week 2 and baseline and (**c**) comparison between week 2 and week 1 (to assess temporal effect)

Lastly, we identified at both the 95% and the 98% similarity level specific OTUs that were significantly affected by raisin intake (Figs. 2 and Fig. 3). At both similarity levels the number of OTUs that differed from baseline was larger in week 1 than week 2 (16 vs. 11 OTUs at 95% similarity and 27 vs. 16 OTUs at 98% similarity level). *Faecalibacterium prausnitzii* significantly increased after the week 1 of raisin intake and this increase continued during the week 2 of raisin consumption (Fig. 3). Multiple OTUs matching Ruminococcaceae as well as *Bacteroidetes spp* significantly increased during both intervention time-periods. Although one OTU matching *Bifidobacterium spp.* significantly decreased with raisin consumption, another OTU classified as *Bifidobacterium longum* increased in week 2 when compared to the week 1. We also observed a significant decrease in an OTU matching *Klebsiella sp.* after week 1 while an OTU matching *Prevotella sp.* decreased after both week 1 and week 2 of raisin consumption.

## Discussion

Dietary intake of sun dried raisins for a continuous period of 14-days did not alter overall microbiota composition but affected the prevalence of OTUs across study participants at various taxon levels. Some of the observed changes are suggestive of potential health benefits. For instance, we observed a significant increase of OTUs matching *Faecalibacterium prausnitzii* after raisin intake. Higher levels of *F. prausnitzii* have been correlated with reduced chronic inflammation and fewer colon polyps [29, 30]. Consistent with our findings, intake of inulin, which is also present in raisins [14], has previously been shown to increase of *F. prausnitzii* levels [31]. Furthermore, the majority of OTUs matching Ruminococcaceae significantly increased during both intervention time-periods as did multiple OTUs matching *Bacteroidetes spp*. Both of these taxa are known to contribute to the degradation of complex carbohydrates resulting in the production of SCFA that contribute to maintaining a balanced gut ecosystem. Our results are consistent with previous studies that reported an increased production of SCFA in fecal samples when raisins are either incubated in vitro or when consumed by humans [12, 15]. We did not detect a consistent increase in Bifidobacteria or Lactic Acid Bacteria, often considered beneficial gut microbes. This could partially be due to amplification bias as Kuczynski et al. reported that 16S rDNA Illumina primers are less efficient in amplifying *Bifidobacterium spp*. [32]. The significant decrease in OTUs matching *Klebsiella sp.,* an opportunistic pathogen, may suggest a reduced risk of subclinical enteric inflammation or reduced potential for urinary tract infections [33–35]. This observation is consistent with previous reports that polyphenolic compounds of grapes can reduce opportunistic pathogens in the gut [36, 37]. While overall we detected no change in *Prevotella sp.* prevalence we observed a specific OTU matching *Prevotella sp.* that decreased during both weeks of raisin consumption. Previous studies have reported an increase in *Prevotella sp.* as a potential health benefit correlating with increased intake of fibers and other plant derived food [38, 39]. While many studies correlate *Prevotella sp.* with improved gut health [4, 40], others suggest that some species such as *Prevotella copri* correlate with chronic inflammation, rheumatoid arthritis, and cardiovascular disease [38, 41, 42]. This example illustrates the difficulty in attributing changes in specific OTUs at the family and even genus level to relevant health benefits.

When compared to baseline, the number of OTUs that significantly changed was greater during the week 1 than week 2. This could indicate that introducing raisins has mostly short-term effects on microbiota. Alternatively, this observation is consistent with reports by participants that indicate reduced compliance during week 2 due to an aversion to consume the required amount of raisins.

Our study was limited by the small number of participants that exhibited a wide range of age, BMI, dietary habits and underlying microbiota composition [43–45]. These large inter-individual variations reduced our power to observe raisin intake associated effects and likely attenuated the true extent of the effect of raisins on gut microbiota. Furthermore, because this was a pilot study with a small sample size we did not adjust for multiple testing in our exploratory analysis. Although we observed several changes in OTUs across study participants who consumed raisins during the study period, without a confirmatory study with a larger sample size these results should be interpreted with caution.

## Conclusions

In this pilot study we observed multiple bacterial taxa that changed in prevalence with the addition of raisins to the diet of healthy volunteers. Broader measures of microbiota diversity were not affected by raisin intake. While the specific OTU’s representing bacterial taxa that changed upon raisin intake in our small study might not be observed in other populations, some OTU level changes likely would occur in other populations. Future studies need to include well-defined health outcomes to establish if the microbial changes resulting from raisin intake correlate with health benefits.

## Additional files

Additional file 1: **Figure S1.** Participant flow chart. (PDF 9 kb)

Additional file 2: **Figure S2.** Chao1 rarefaction diversity (α-diversity, *N* = 13). (PDF 110 kb)

Additional file 3: **Figure S3.** UniFrac beta diversity analysis (*N* = 13). (PDF 48 kb)

## Abbreviations

BLAST: Basic Local Alignment Tool
OTUs: Operational Taxonomic Units
QIIME: Quantitative Insights into Microbial Ecology
RDP: Ribosomal Database Project
SCFAs: Short Chain Fatty Acids
