# Supplementary figures and images for: Dietary raisin intake has limited effect on gut microbiota composition in adult volunteers
Source: Nutr J. 2019 Mar 7;18:14. doi: 10.1186/s12937-019-0439-1 (PMC6404294; doi:10.1186/s12937-019-0439-1)

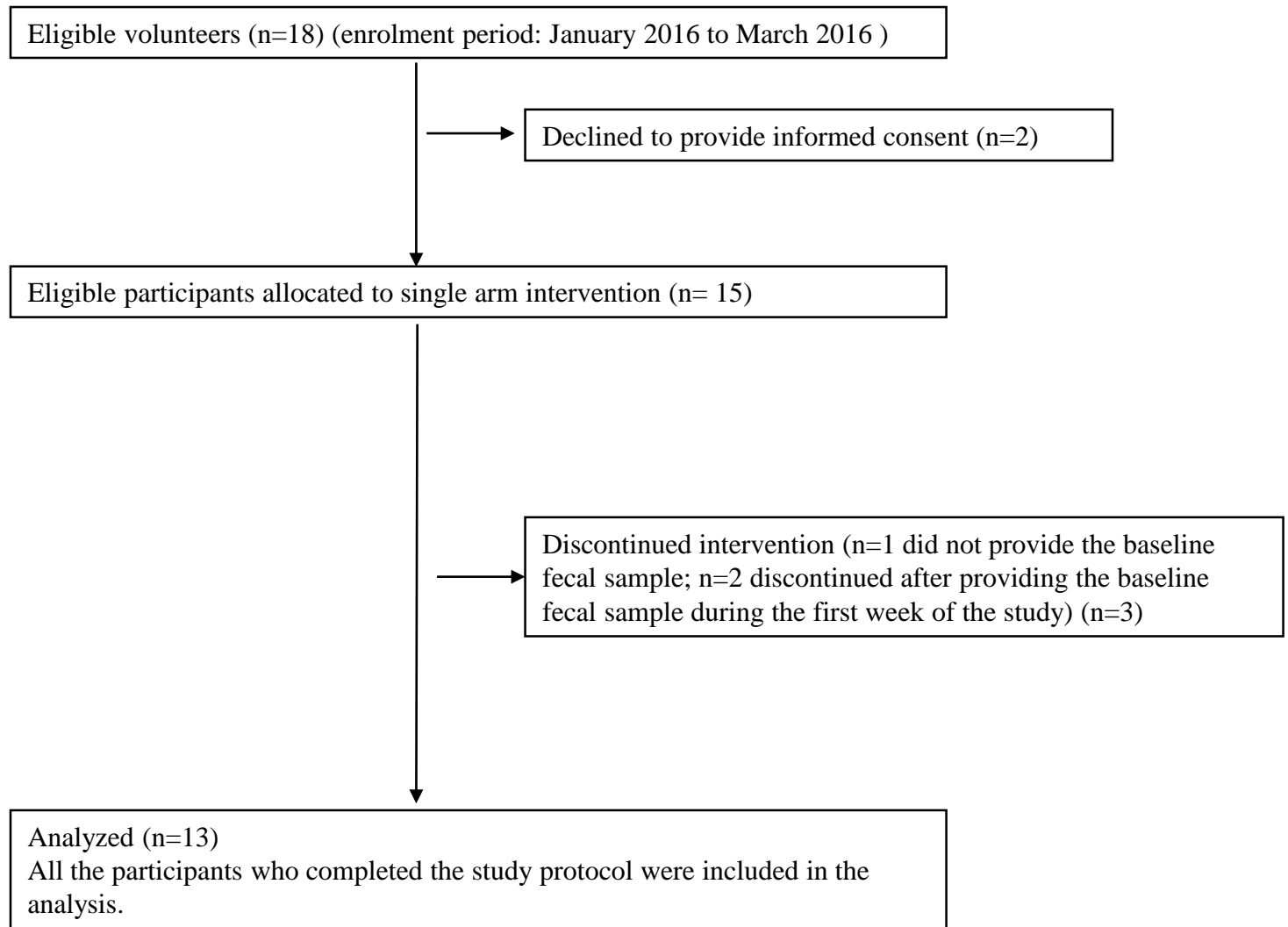

**Figure S1.** Participant flow chart.

Supplement: Supplementary file 1 — Figure S1. Participant flow chart. (PDF 9 kb) [file 12937_2019_439_MOESM1_ESM.pdf]
