# Supplementary material for: Dietary raisin intake has limited effect on gut microbiota composition in adult volunteers
Source: Nutr J. 2019 Mar 7;18:14. doi: 10.1186/s12937-019-0439-1 (PMC6404294; doi:10.1186/s12937-019-0439-1)
Supplement: Supplementary file 2 — Figure S2. Chao1 rarefaction diversity (α-diversity, N = 13). (PDF 110 kb) [file 12937_2019_439_MOESM2_ESM.pdf]

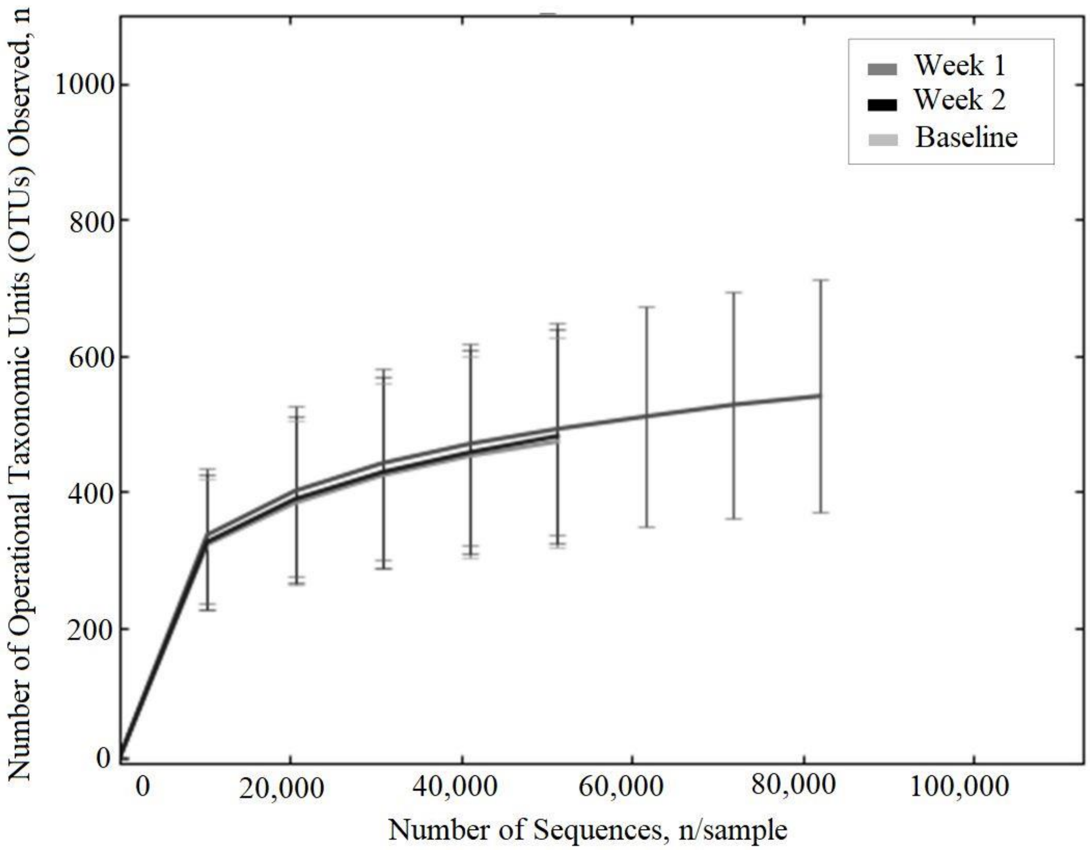

Figure S1. Chao1 rarefaction diversity (α-diversity, N=13). Chao diversity was calculated from the 16S rRNA gene sequence distribution at baseline, week 1 (first week of continuous raisin intake) and week 2 (second week of raisin intake).
