# Supplementary material for: Dietary raisin intake has limited effect on gut microbiota composition in adult volunteers
Source: Nutr J. 2019 Mar 7;18:14. doi: 10.1186/s12937-019-0439-1 (PMC6404294; doi:10.1186/s12937-019-0439-1)
Supplement: Supplementary file 3 — Figure S3. UniFrac beta diversity analysis (N = 13). (PDF 48 kb) [file 12937_2019_439_MOESM3_ESM.pdf]

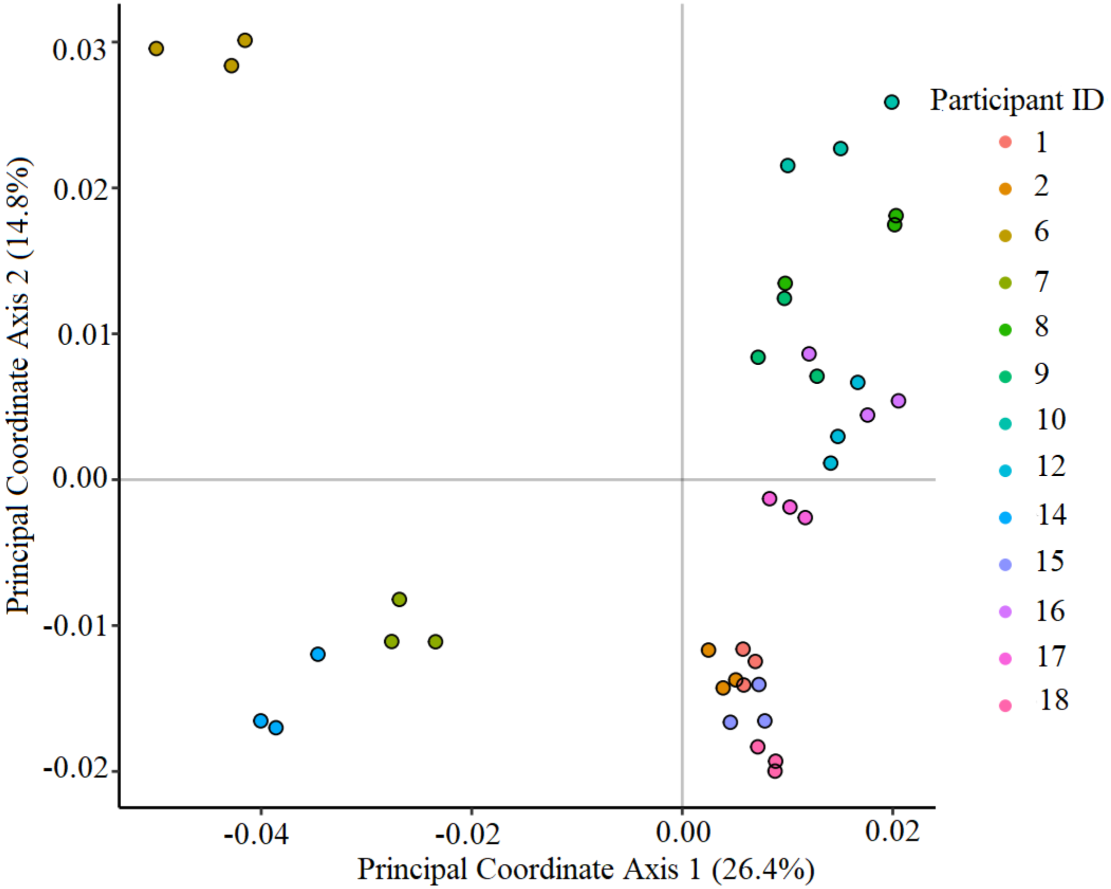

Figure S2. UniFrac beta diversity analysis (N=13). The principle coordinate plot based on weighted UniFrac distances presents microbiota composition in fecal samples measured at baseline, week 1 (one week after starting raisin intake) and week 2 (two weeks after raisin consumption). Samples from each study participant are color coded.
